# Systematic STI screening in unaccompanied migrant minors: a 6-year experience with urine-based NAAT

**DOI:** 10.1007/s00431-026-07549-3

**Published:** 2026-10-26

**Authors:** Sonia Milkova-Ivanova, Isabel Mellado-Sola, Blanca Bravo-Queipo-de-Llano, Paula Rodríguez-Molino, Carlos Grasa, Milagros García-López-Hortelano, Inmaculada Quiles-Melero, Mario David Ruiz-Bastián, Alfredo Maldonado-Barrueco, Cristina Calvo, Talía Sainz

**Affiliations:** 1 Pediatric, Infectious and Tropical Diseases Department, La Paz University Hospital, La Paz Research Institute (IdiPAZ), Paseo de La Castellana 261, 28046 Madrid, Spain; 2 Autonomous University of Madrid (UAM), Madrid, Spain; 3 Networked Biomedical Research Centre for Infectious Diseases (CIBERINFEC), Carlos III Health Institute, Madrid, Spain; 4 Clinical Microbiology Department, La Paz University Hospital, La Paz Research Institute (IdiPAZ), Madrid, Spain

**Keywords:** Unaccompanied migrant minors, Sexually transmitted infections, Screening, Nucleic acid amplification tests

## Abstract

Systematic screening for sexually transmitted infections (STI), beyond serology, is not standard in arrival protocols for unaccompanied migrant minors (UMM). In 2020, our center incorporated urine-based nucleic acid amplification tests (NAAT)-based screening into the existing serology-based protocol (HIV, syphilis, hepatitis B/C), and we describe the additional diagnoses of STI identified through NAAT. We conducted a retrospective observational descriptive study (2020–2025) of 226 UMM (99.6% male) in Madrid undergoing serological screening for HIV, syphilis, hepatitis B and C, with additional urine-based multiplex PCR (NAAT) screening for STI. Urine-based NAAT, performed in 163/226 participants (72.1%), identified six positive cases (3.7%, 95% CI 1.4–7.8%) of *C. trachomatis*, *M. genitalium*, or *Ureaplasma* spp. Of these, four patients (2.5%, 95% CI 0.7–6.2%) had actionable infections that were treated. Serology identified no HIV (0/218) or hepatitis C cases (0/212), one syphilis case (0.5%, 95% CI 0.0–2.6%, 1/209), and three chronic hepatitis B cases (1.4%, 95% CI 0.3–3.9%, 3/221). None of the cases had symptoms recorded.

*Conclusion*: Urine NAAT identified six infections (four actionable) not detectable by serology. However, three of eight patients with actionable infections were lost to follow-up, highlighting that the benefit of systematic screening depends on strengthening linkage-to-care alongside testing.

**Table:** 

**What is Known:** • *Serological screening (HIV, syphilis, hepatitis B/C) is standard in arrival health assessments for unaccompanied migrant minors, but systematic STI screening beyond serology is not routinely protocoled.* • *Systematic screening is especially valuable in this population, as follow-up is often limited by care fragmentation and transfers between reception centers.*	
**What is New:** • *Urine-based NAAT screening identified additional STI in patients with no symptoms recorded, that were not detectable by serology alone.*

**What is Known:**

• *Serological screening (HIV, syphilis, hepatitis B/C) is standard in arrival health assessments for unaccompanied migrant minors, but systematic STI screening beyond serology is not routinely protocoled.*

• *Systematic screening is especially valuable in this population, as follow-up is often limited by care fragmentation and transfers between reception centers.*

**What is New:**

• *Urine-based NAAT screening identified additional STI in patients with no symptoms recorded, that were not detectable by serology alone.*

## Introduction

Unaccompanied migrant minors (UMM) are a vulnerable population exposed to multiple health risks and facing barriers to healthcare access, including linguistic and cultural obstacles and frequent transfers between reception centers, which complicate follow-up and continuity of care. Even when infections are diagnosed, high rates of follow-up loss remain a major concern.

Spain has experienced an increase in arriving UMM in recent years, with 16,041 registered in 2024, the majority originating from Morocco, Senegal, and Algeria [1]. Current guidelines recommend routine health assessments upon arrival, including serological screening for HIV, hepatitis, and syphilis [2], but systematic sexually transmitted infections (STI) screening beyond serology is not universally recommended, and practices vary across regions [3].

This gap contrasts with the burden of STI among adolescents: in the EU, adolescents aged 15–19 years accounted for 16% of notified *Chlamydia* cases in 2024 [4]. WHO recommends increased use of non-invasive nucleic acid amplification tests (NAAT) for STI screening [5].

In Spain, national surveillance data reported an incidence of *C. trachomatis* infection of 86.26 cases per 100,000 inhabitants in the general population (all ages) in 2024, with adolescents aged 15–19 years accounting for 9.7% of notified cases with available age and sex data (4073/41,911) [6]. However, data on STI prevalence in UMM specifically remain scarce. Studies in adult refugees and asylum seekers describe a substantial prevalence of HIV, syphilis, and hepatitis B and C, with regional variation by country of origin [3].

In 2020, our center incorporated systematic urine-based NAAT screening for STI into the existing UMM arrival protocol, given the lack of evidence on STI detectable by NAAT in this population, which are not captured by serology. We report here our 6-year experience addressing this specific question.

## Methods

We conducted a retrospective observational study with a descriptive design including UMM under 18 years of age attended at a pediatric infectious disease unit of a tertiary referral hospital in Madrid, Spain, between January 2020 and November 2025. Inclusion criteria were age under 18, unaccompanied migrant status, and availability of a urine and/or serum sample. Most patients, based on clinical records, were assessed within the first year after arrival in Spain, although referral timing varied and exact arrival dates were not systematically recorded in all patients.

The multiplex PCR Allplex™ STI Essential Assay (Seegene®, Seoul, South Korea) detected *Chlamydia trachomatis*, *Mycoplasma genitalium*, *Mycoplasma hominis*, *Neisseria gonorrhoeae*, *Trichomonas vaginalis*, *Ureaplasma parvum*, and *Ureaplasma urealyticum*. Multiplex real-time PCR assays, including the Allplex™ platform, have shown good clinical performance for *C. trachomatis* and *N. gonorrhoeae* detection specifically in first-void urine samples [7]. Urine samples were collected first void when possible; collection conditions were not standardized given the retrospective design. Serological screening for HIV, syphilis, and hepatitis B and C was performed as part of the existing protocol. Chronic hepatitis B infection was defined as persistent HBsAg positivity for more than 6 months, confirmed by repeat serological testing [8]. Patients were considered to have an actionable infection when, based on clinical guidelines and medical judgment, treatment or specialist follow-up was indicated. Not all participants had complete testing due to sample unavailability or loss to follow-up before workup completion. Demographic, clinical (including symptoms), and laboratory data were extracted from medical records. Follow-up was considered confirmed when treatment was completed or the patient remained under documented follow-up.

The available information was analyzed by descriptive statistics using the IBM SPSS version 26 software. Tested and untested participants were compared using chi-square or Fisher’s exact test for categorical variables and the Mann–Whitney *U* test for age. All data were included in an anonymized dataset. The study was approved by the hospital’s Ethics Committee (PI-3348).

## Results

The cohort included 226 UMM, almost exclusively male and originating from Morocco and Sub-Saharan Africa (Table 1).

**Table 1:** Baseline characteristics of the study population

Category	Parameter	Overall population (*N* = 226)	95% CI
**Demographics**	Age, years, median (IQR)	17 (16.4–17.6)	
	Male sex, *n* (%)	225 (99.6)	
	Country of origin, *n* (%)		
	Morocco	96 (42.5)	
	Gambia	47 (20.8)	
	Senegal	26 (11.5)	
	Mali	18 (8.0)	
	Other*	39 (17.3)	
**STI serology**	HIV, *n*/*N* (%)	0/218 (0)	0.0–1.7%
	Syphilis, *n*/*N* (%)	1/209 (0.5)	0.0–2.6%
	Chronic hepatitis B (HBsAg +), *n*/*N* (%)	3/221 (1.4)	0.3–3.9%
	Hepatitis C, *n*/*N* (%)	0/212 (0)	0.0–1.7%
**STI urine-based NAAT**	Assessed, *n*/*N* (%)	163/226 (72.1)	65.8–77.9%
	Positive, *n*/*N* (%)	6/163 (3.7)	1.4–7.8%
	Positive, treated, *n*/*N* (%)	4/163 (2.5)	0.7–6.2%
**Follow-up among patients with actionable infections**^*†*^	Lost to follow up, *n*/*N* (%)	3/8 (37.5)	8.5–75.5%
	Confirmed follow up, *n*/*N* (%)	5/8 (62.5)	24.5–91.5%

Data are presented as median (IQR), *n* or *n*/*N* (%), as appropriate. Denominators vary due to missing data

^*^Other countries (< 5% each): Guinea-Conakry, Algeria, Cameroon, Côte d’Ivoire, Guinea-Bissau, Somalia, Nigeria, Bangladesh, Mauritania, Angola, Zambia

^†^Patients with actionable infections: treated NAAT-positive cases (*n* = 4), syphilis (*n* = 1) and chronic hepatitis B (*n* = 3). The two untreated NAAT-positive cases (one *Ureaplasma* spp., one *M. genitalium*) are excluded

Overall, 10 positive results were identified in this cohort, six by NAAT and four by serology, all of them in patients with no symptoms recorded.

Urine-based NAAT screening for STI was performed in 72.1% (163/226) of individuals, all male. Six patients (3.7%, 95% CI 1.4–7.8%) tested positive: two (1.2%, 95% CI 0.1–4.4%) for *C. trachomatis* alone, two (1.2%, 95% CI 0.1–4.4%) for *M. genitalium* alone, one (0.6%, 95% CI 0.0–3.4%) for *C. trachomatis* and *M. genitalium* in coinfection, and one (0.6%, 95% CI 0.0–3.4%) for *Ureaplasma* spp. None had symptoms recorded. Four completed treatments at our center (2.5%, 95% CI 0.7–6.2%, considered actionable infections): the three *C. trachomatis* infections, including the coinfection, and one *M. genitalium* monoinfection, which was treated because of a reported risk exposure. The remaining two (one *Ureaplasma* spp. and one *M. genitalium* monoinfection without reported risk exposure) were not treated, based on clinical judgment, and were therefore not considered actionable. No cases of *N. gonorrhoeae* or *T. vaginalis* were detected among the 163 individuals tested. Among NAAT-positive cases, the interval between arrival in Spain and diagnosis ranged from 1 to 18 months (median 4.5 months); the longest interval reflected a prolonged itinerary through multiple reception points before referral to our center.

Serological testing confirmed that no participants were positive for HIV or hepatitis C. One case of syphilis and three cases of chronic hepatitis B were detected, with chronicity confirmed by repeat HBsAg testing beyond 6 months. None of the participants with syphilis or hepatitis B had symptoms recorded. The syphilis case completed treatment at our center. The three hepatitis B cases were subsequently lost to follow-up, one of them after referral to adult gastroenterology services.

We compared tested and untested participants for NAAT and serology. Age did not differ significantly in any comparison (*p* > 0.3). Country of origin showed a borderline association for NAAT (*p* = 0.056), with Moroccan-origin children overrepresented among the untested (57.1% vs. 36.8%) and Gambian-origin children underrepresented (11.1% vs. 24.5%). No such association was found for the serologies (all *p* > 0.4).

Overall, three of the eight patients with actionable infections (37.5%) were lost to follow-up, all of them chronic hepatitis B cases (Table 1).

## Discussion

In this cohort of UMM, all positive results detected by either NAAT or serology occurred in patients with no recorded symptoms. However, as symptom status relied on retrospective documentation, we cannot establish whether symptom-based testing would have identified these cases. The additional diagnostic yield of urine NAAT beyond serology was 6/163 (3.7%) positive results, four of them actionable (2.5%).

The prevalence of STI detected by urine-based NAAT in patients with no symptoms recorded was lower than reported using the same panel in a Spanish population of sexually active young adults (11.4%), driven mainly by *C. trachomatis* (7.4%) and *U. urealyticum (*3.7%) [9]. However, this comparison is limited by an age difference (18–25 years) vs. our cohort’s median age of 17. A more age-matched comparator is a UK cohort of unaccompanied asylum-seeking children of similar median age using the same NAAT methodology, which found a *Chlamydia* prevalence of 0.7% (1/148), which falls within the confidence interval of our *C. trachomatis* estimate (3/163, 1.8%, 95% CI 0.4–5.3%), and a syphilis prevalence of 2.0% (3/152) [10], also within our own pathogen-specific confidence intervals.

Systematic urine-based NAAT STI screening is not yet routinely included in health arrival protocols for newly arrived UMM, although our findings suggest that it may be a valuable addition. Most comparable cohorts of UMM report screening limited to HIV, hepatitis B/C, and syphilis serology [11], with NAAT-detected pathogens such as *C. trachomatis* rarely incorporated systematically. The 2025 WHO guidelines on the management of asymptomatic STI restrict their screening recommendations to *N. gonorrhoeae* and *C. trachomatis*, in defined populations and only where prevalence is high [5]. *Ureaplasma* spp. And *M. hominis*, also detected by our multiplex panel, are not listed among WHO’s screening targets, consistent with their status as commensals rather than established STI pathogens. By contrast, *M. genitalium* is an established sexually transmitted pathogen, but it is not among WHO’s screening targets either [5], and current guidelines advise against screening asymptomatic individuals given the lack of evidence of benefit and concerns about antimicrobial resistance [12]. The management of asymptomatic *M. genitalium* detected incidentally by multiplex panels is not standardized; in our cohort, treatment decisions were individualized according to reported risk exposure, although, as discussed below, reliance on disclosed risk may be limited in this population. Our focus on actionable infections is therefore aligned with current guidance.

Based on 4 patients with actionable infections identified among 163 individuals tested, the number needed to screen was approximately 41 (95% CI 16–149), reflecting the imprecision inherent to the small number of events. This should be weighed against the cost of the multiplex NAAT panel per sample when considering implementation in routine arrival protocols. At our center, screening this cohort cost approximately 3260€ in total, or 815€ per patient with actionable infection detected (20€/sample). Whether this cost is justified depends on local budget constraints and the downstream costs averted by early treatment (e.g., pelvic inflammatory disease, infertility), which were not evaluated in this study. Because urine-based NAAT would be added to an entry health assessment where serological screening infrastructure (HIV, hepatitis B/C, syphilis) and staff time are already funded, the marginal cost of incorporating NAAT into that existing pathway—rather than the cost of a stand-alone program—is the relevant figure for policy-makers, and we identify this as a priority for future prospective research before wider adoption.

However, a risk-based approach, reserving testing for individuals presenting with symptoms or disclosing high-risk behaviors is controversial in adolescent medicine, and may be fundamentally inadequate for UMM. This population faces unique vulnerabilities throughout the migratory process, including potential exposure to sexual violence and limited access to preventive healthcare [13]. Linguistic barriers, cultural taboos, and confidentiality issues further limit the reliability of symptom or disclosure-based screening in this group. If left untreated, STI can lead to long-term complications, including pelvic inflammatory disease and infertility [8].

Our findings are consistent with the use of urine-based NAAT for targeted *C. trachomatis* and *N. gonorrhoeae* testing in asymptomatic individuals younger than 25 years of age [9]. This approach is non-invasive, has shown good performance in urine samples [7], and is well tolerated: an important consideration in a population where minimizing unnecessary medical procedures helps avoid re-victimization. Routine screening for the other organisms in the panel is not supported by current guidance [5, 12], and their incidental detection raises unresolved management questions. In our cohort, most actionable infections detected by NAAT were *C. trachomatis* (3/4), while the remaining organisms contributed little additional actionable yield.

Three of the eight patients with actionable infections were lost to follow-up, all of them chronic hepatitis B cases, one after transfer to adult gastroenterology services. Care fragmentation in this population is not only geographic, driven by transfers between reception centers, but also structural: transitions between pediatric and adult care, limited availability of intercultural mediators, and restricted access to prior medical records all hinder continuity of care. Given these barriers, every clinical encounter may be the only realistic opportunity for screening and linkage to care.

This study is limited by its single-center, retrospective design, small sample size, and near-exclusive male composition, reflecting the demographic profile of UMM arriving in Spain during this period; findings may not generalize to female or mixed-gender cohorts. In addition, our cohort was restricted to a narrow age range (median 17 years, IQR 16.4–17.6), corresponding to older adolescents (15–19 years per national surveillance age-group categories [6]). Our findings and recommendations therefore apply specifically to this age group and should not be extrapolated to younger children or to UMM populations with broader age distributions. To assess selection bias, we compared tested and untested participants for NAAT and serology; country origin showed a borderline difference for NAAT, so our estimates may be less representative of Moroccan-origin participants.

The inability to confirm follow-up outcomes is another limitation, highlighting the critical challenge in ensuring continuity of care for this population. Besides, the absence of extra-genital (rectal, pharyngeal) sampling may have underestimated the true prevalence of some pathogens. Also, as the exact date of arrival in Spain was not systematically recorded for the entire cohort, arrival-to-consultation intervals could only be calculated for NAAT-positive cases. Finally, symptom status was based on the absence of documented symptoms in the clinical record; given the linguistic barriers, cultural taboos and confidentiality concerns described above, under-reporting of symptoms cannot be excluded and may have led to an overestimation of the proportion of infections classified as asymptomatic.

In conclusion, all positive findings had no recorded symptoms, and urine NAAT identified infections not detectable by serology. However, three of the eight patients with actionable infections in our cohort were lost to follow-up. These findings suggest that urine NAAT-based STI screening, with targets guided by current guidance, may be a valuable addition to the initial health assessment of newly arrived UMM with a demographic similar to ours -almost exclusively male-, provided it is paired with stronger linkage-to-care strategies; without this, screening may identify infections without improving clinical outcomes.

## Data Availability

The data that support the findings of this study are available from the corresponding author upon reasonable request.
